# Supplementary material for: Comprehensive analysis of cis- and trans-acting factors affecting ectopic Break-Induced Replication
Source: PLoS Genet. 2022 Jun 21;18(6):e1010124. doi: 10.1371/journal.pgen.1010124 (PMC9249352; doi:10.1371/journal.pgen.1010124)
Supplement: S1 Fig — Combinations of two exponential functions are presented, with the first function fitting the data either from IVR-10 to IVR-26, or from IVR-10 to IVR-36, or from IVR-10 to IVR-41, or from IVR-10 to IVR-46. (PDF) [file pgen.1010124.s001.pdf]

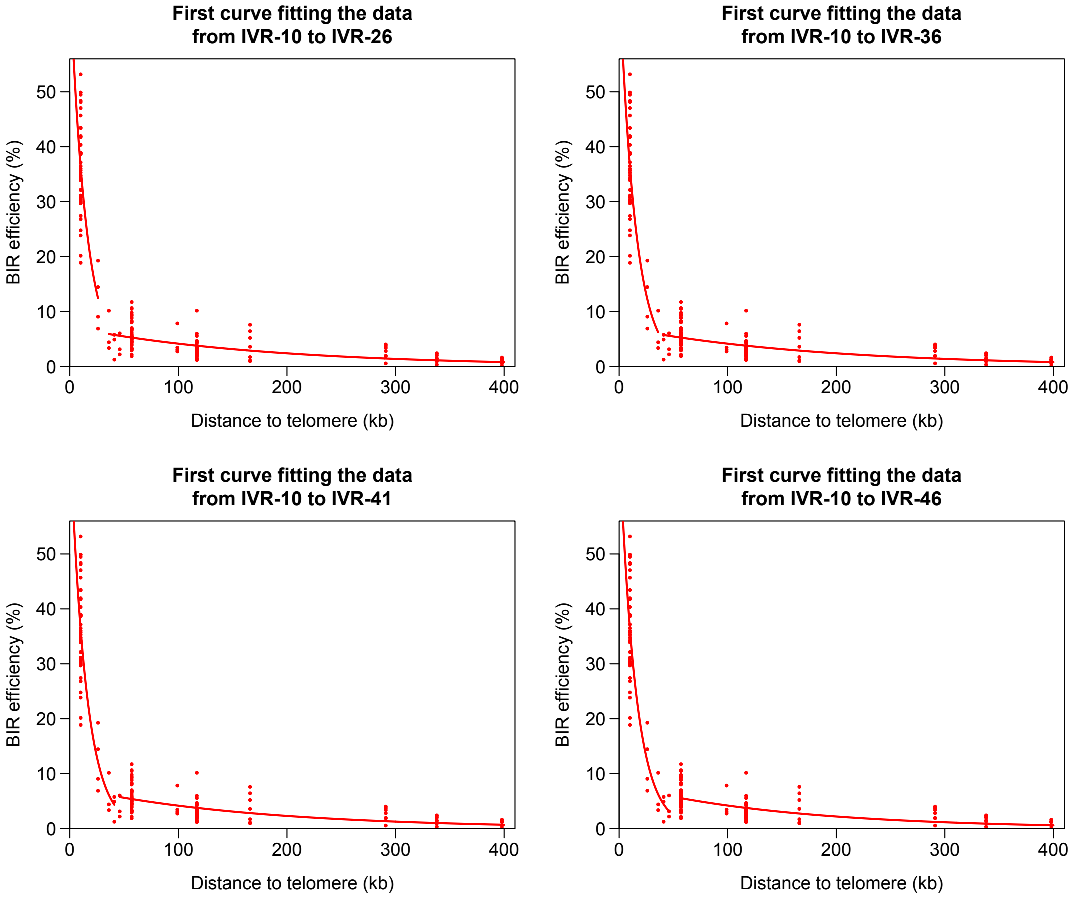

S1 Fig: Fitting the relationship between BIR efficiency and the length of DNA to synthesize. Combinations of two exponential functions are presented, with the first function fitting the data either from IVR-10 to IVR-26, or from IVR-10 to IVR-36, or from IVR-10 to IVR-41, or from IVR-10 to IVR-46.
