# Supplementary material for: Comprehensive analysis of cis- and trans-acting factors affecting ectopic Break-Induced Replication
Source: PLoS Genet. 2022 Jun 21;18(6):e1010124. doi: 10.1371/journal.pgen.1010124 (PMC9249352; doi:10.1371/journal.pgen.1010124)
Supplement: S2 Fig — Note that the pADW17 plasmid containing a telomeric seed was used as a transformation control for the pol32 mutant but could not be used in the pif1m2 mutants. Instead, the pRS416 plasmid was used. The transformation efficiency of pRS416 is higher than the transformation efficiency of pADW17. This likely explains the difference in BIR efficiency between the WT strains from the two graphs. Indicated are Student’s t-test p-values <0.05 when comparing BIR efficiency from a given locus with its equivalent from the corresponding WT. WT values from the left graph are the same as in Fig 3 and represent the means of 49 independent experiments. The pol32 values from the left graph are the means of three independent experiments. WT values from the right graph are the means of four independent experiments, and mutant values from the right graph are the means of three independent experiments. Error bars represent standard deviations. (PDF) [file pgen.1010124.s002.pdf]

A.

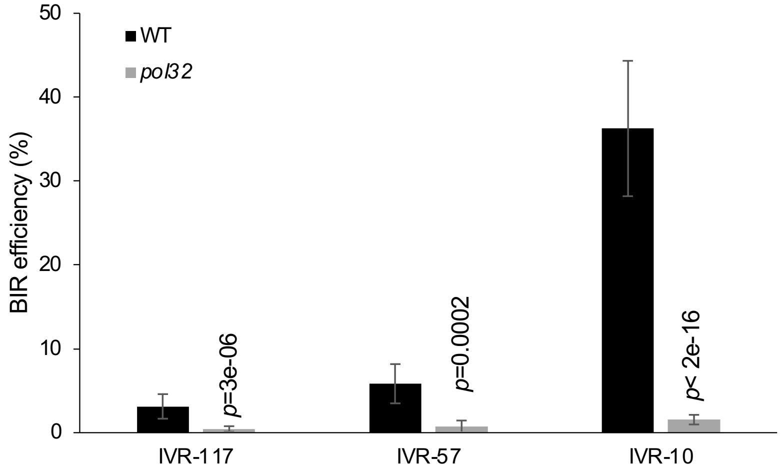

B.

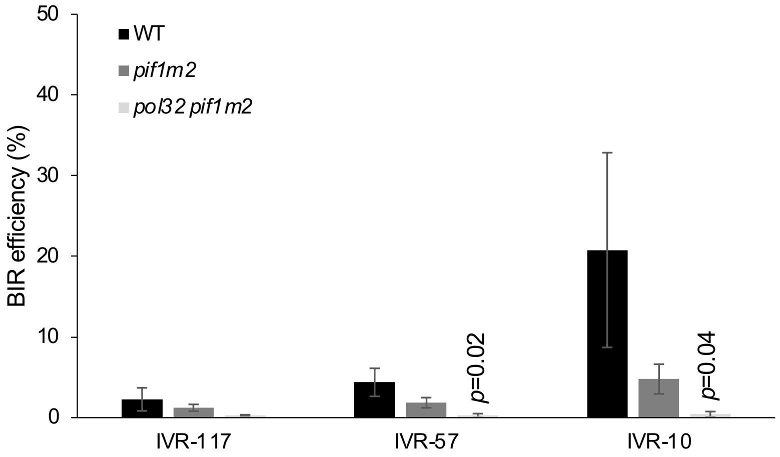

S2 Fig: BIR efficiency in *pol32* (**panel A**) and *pif1m2* (**panel B**) mutants. Note that the pADW17 plasmid containing a telomeric seed was used as a transformation control for the *pol32* mutant but could not be used in the *pif1m2* mutants. Instead, the pRS416 plasmid was used. The transformation efficiency of pRS416 is higher than the transformation efficiency of pADW17. This likely explains the difference in BIR efficiency between the WT strains from the two graphs. Indicated are Student's *t*-test *p*-values <0.05 when comparing BIR efficiency from a given locus with its equivalent from the corresponding WT. WT values from the left graph are the same as in Figure 3 and represent the means of 49 independent experiments. The *pol32* values from the left graph are the means of three independent experiments. WT values from the right graph are the means of four independent experiments, and mutant values from the right graph are the means of three independent experiments. Error bars represent standard deviations.
