# Supplementary material for: Comprehensive analysis of cis- and trans-acting factors affecting ectopic Break-Induced Replication
Source: PLoS Genet. 2022 Jun 21;18(6):e1010124. doi: 10.1371/journal.pgen.1010124 (PMC9249352; doi:10.1371/journal.pgen.1010124)

S3 Fig: PFGE analysis of BIR products. Individual transformants were used to prepare DNA plugs that were analyzed by PFGE. Each gel lane corresponds to a unique transformant. Ethidium bromide stained gels (left) and corresponding radioactive signal from Southern blots using a *URA3* probe specific of the chromosome fragmentation vector (right) are shown. Arrowheads indicate chromosome fragments (CF) of abnormal size resulting from BIR initiated by the chromosome fragmentation vector. Gross chromosomal rearrangements (GCR) are indicated by arrows. **A**. Reference strain BY4741 transformed with the chromosome fragmentation vector initiating BIR at the IVR-10 locus. **B**. Reference strain BY4741 transformed with the chromosome fragmentation vector initiating BIR at the IVR-117 locus. **C**. Translocated IVR-113_XVI-12 strain transformed with the chromosome fragmentation vector initiating BIR at the IVR-117 locus. Note that chromosome IV from this strain is 101 kb shorter than its normal size. This is translated by a longer migration in the gel and a longer distance from chromosome XII as compared to all PFGE using non-translocated strains. **D**. *srs2* null strain transformed with the chromosome fragmentation vector initiating BIR at the IVR-10 locus. Note that GCRs involving chromosome XII that contains the rDNA locus were not considered. **E**. *srs2* null strain transformed with the circular chromosome fragmentation vector. Ethidium bromide stained gels only are shown since no BIR takes place in this context. The rationale of this experiment was to test for potential GCR in the absence of Srs2. No GCR were detected out of 100 clones.

A.

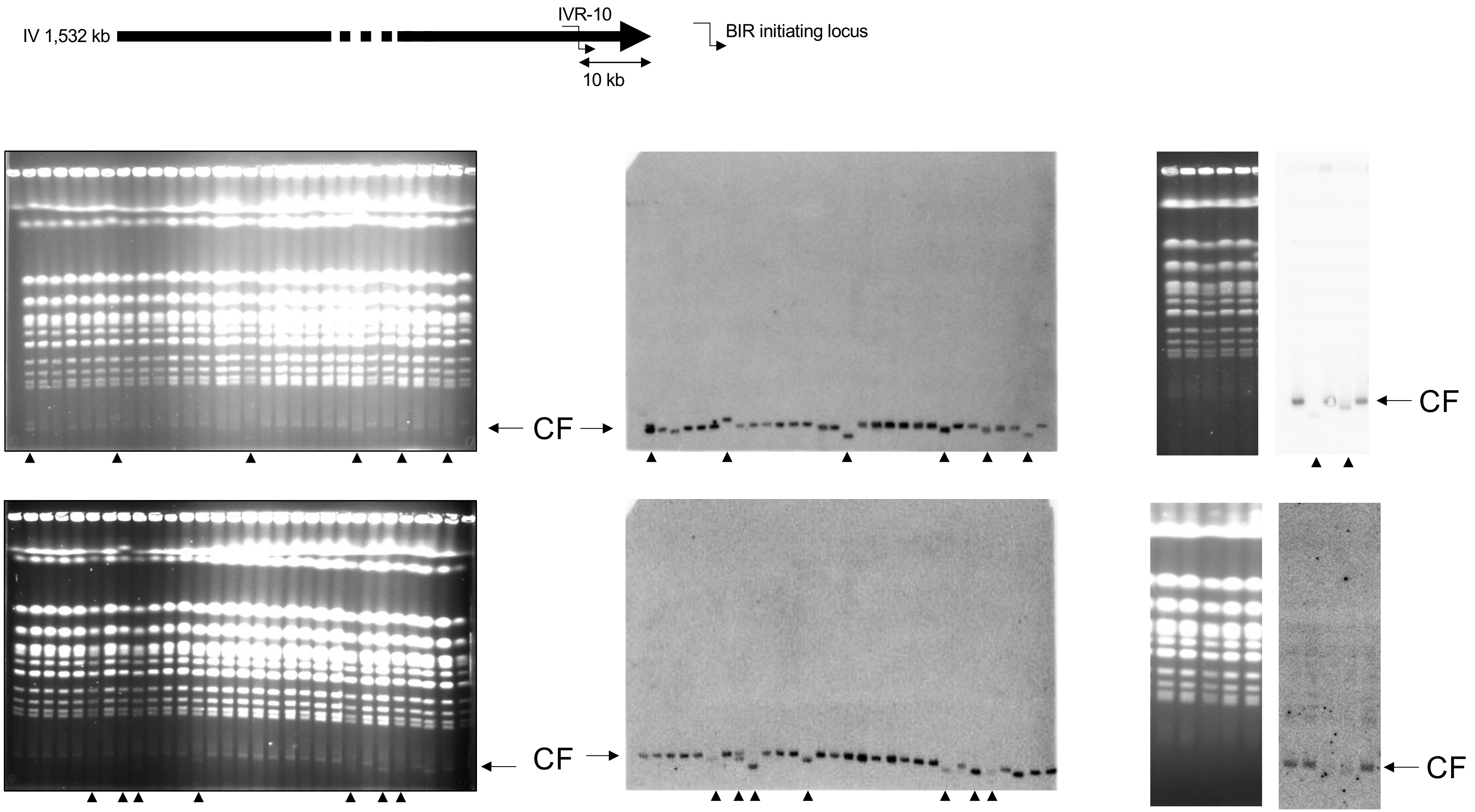

B.

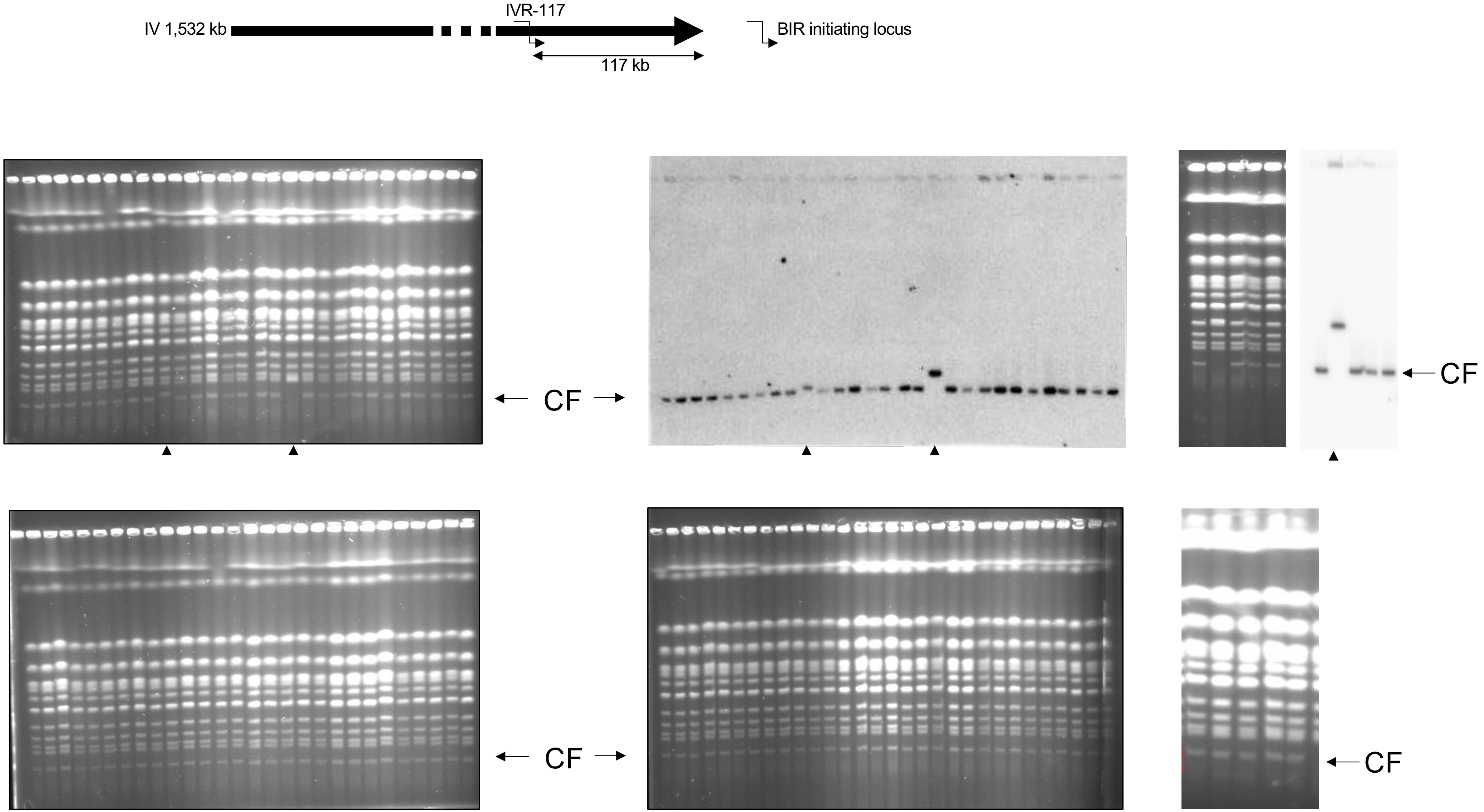

C.

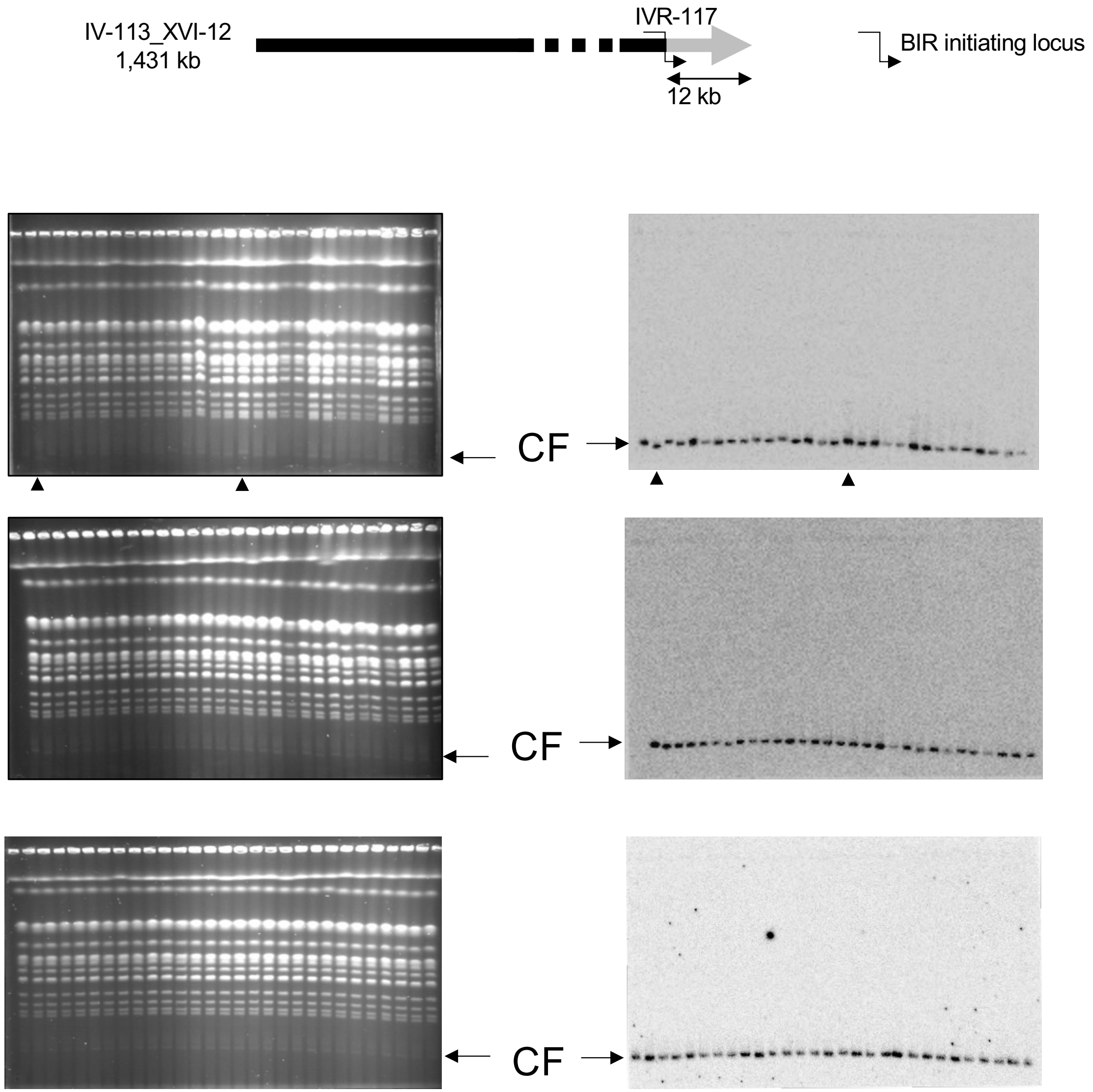
IV-113_XVI-12
1,431 kb
IVR-117
12 kb
BIR initiating locus
CF
CF
CF

D.

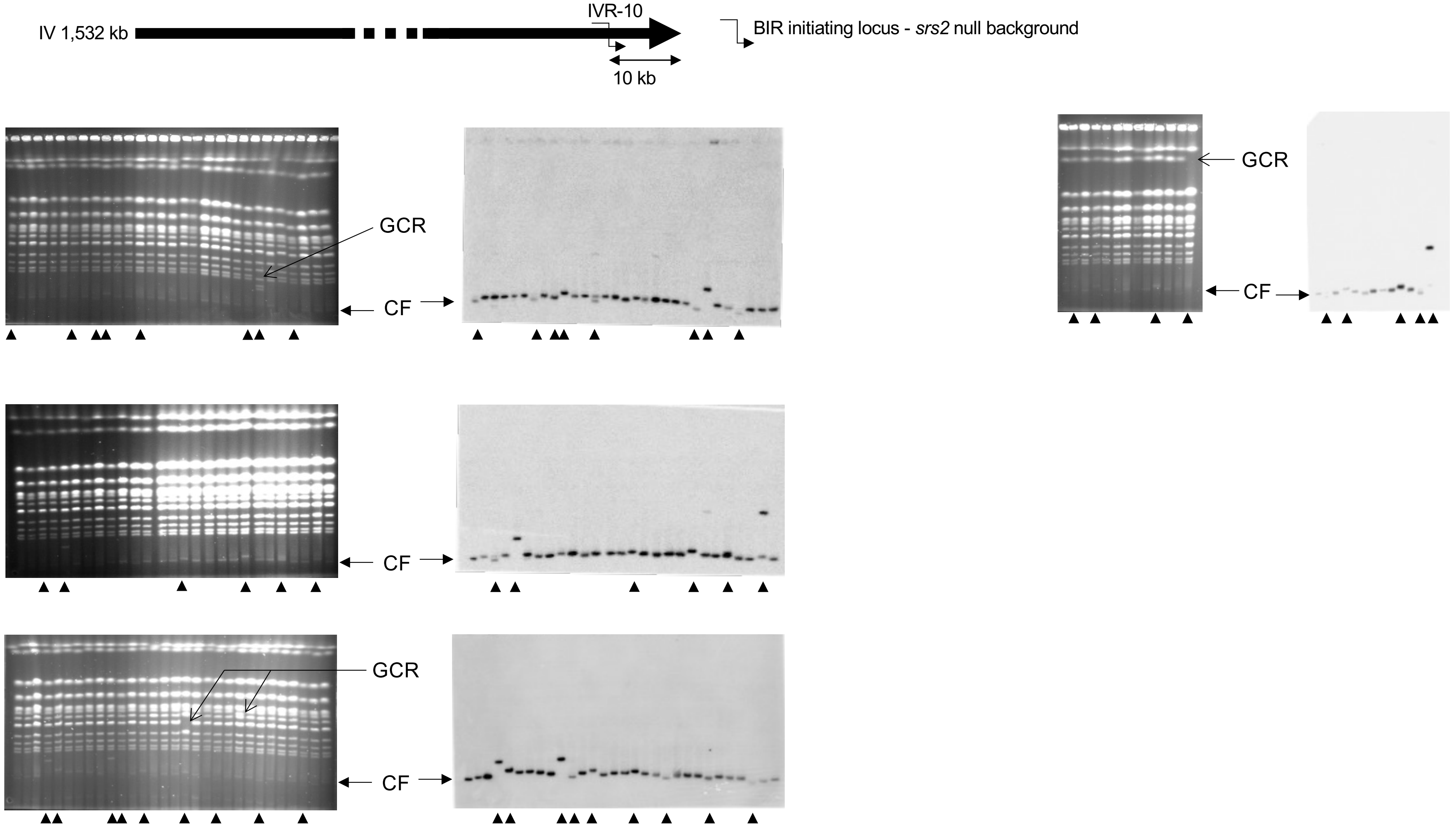
IVR-10
IV 1,532 kb
BIR initiating locus - srs2 null background
10 kb
GCR
CF
GCR
CF
CF
GCR
CF

E.

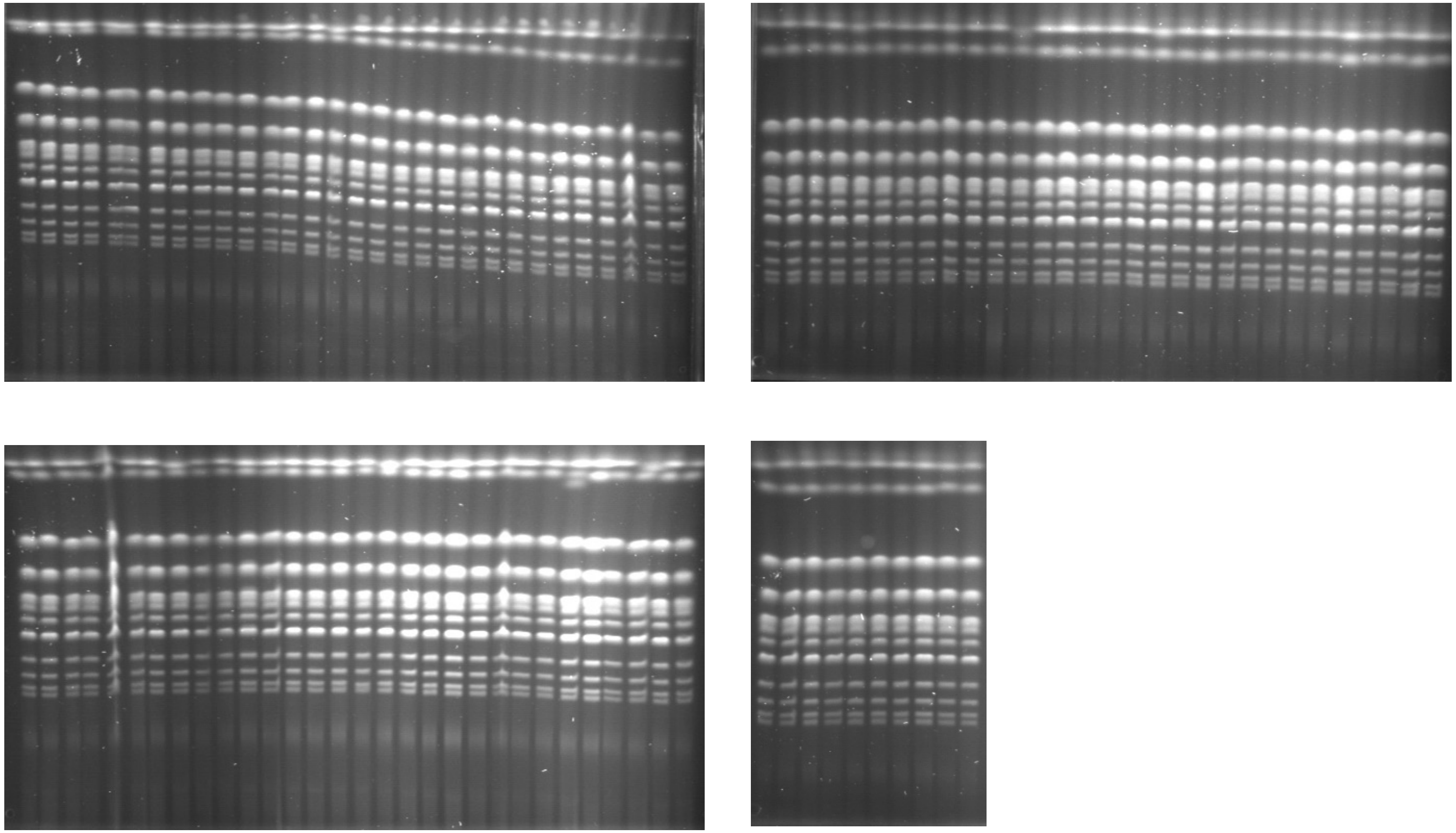

Supplement: S3 Fig — Individual transformants were used to prepare DNA plugs that were analyzed by PFGE. Each gel lane corresponds to a unique transformant. Ethidium bromide stained gels (left) and corresponding radioactive signal from Southern blots using a URA3 probe specific of the chromosome fragmentation vector (right) are shown. Arrowheads indicate chromosome fragments (CF) of abnormal size resulting from BIR initiated by the chromosome fragmentation vector. Gross chromosomal rearrangements (GCR) are indicated by arrows. A. Reference strain BY4741 transformed with the chromosome fragmentation vector initiating BIR at the IVR-10 locus. B. Reference strain BY4741 transformed with the chromosome fragmentation vector initiating BIR at the IVR-117 locus. C. Translocated IVR-113_XVI-12 strain transformed with the chromosome fragmentation vector initiating BIR at the IVR-117 locus. Note that chromosome IV from this strain is 101 kb shorter than its normal size. This is translated by a longer migration in the gel and a longer distance from chromosome XII as compared to all PFGE using non- translocated strains. D. srs2 null strain transformed with the chromosome fragmentation vector initiating BIR at the IVR-10 locus. Note that GCRs involving chromosome XII that contains the rDNA locus were not considered. E. srs2 null strain transformed with the circular chromosome fragmentation vector. Ethidium bromide stained gels only are shown since no BIR takes place in this context. The rationale of this experiment was to test for potential GCR in the absence of Srs2. No GCR were detected out of 100 clones. (PDF) [file pgen.1010124.s003.pdf]
