# Supplementary material for: Comprehensive analysis of cis- and trans-acting factors affecting ectopic Break-Induced Replication
Source: PLoS Genet. 2022 Jun 21;18(6):e1010124. doi: 10.1371/journal.pgen.1010124 (PMC9249352; doi:10.1371/journal.pgen.1010124)
Supplement: S4 Fig — A. Relates to Fig 4A. IVR-10_VIL-16 is the translocated strain containing THI5 translocated downstream of the IVR-10 locus. Mean ratios of THI5 and ACT1 RT-qPCR quantifications from two independent experiments are indicated. Error bars indicate the deviation from the mean. B. Relates to Fig 5B. Ratios of RAD51 and RAD52 RT-qPCR quantifications normalized by the RT-qPCR quantification of ACT1. Overexpression of RAD51 and RAD52 was verified with one experiment only. (PDF) [file pgen.1010124.s004.pdf]

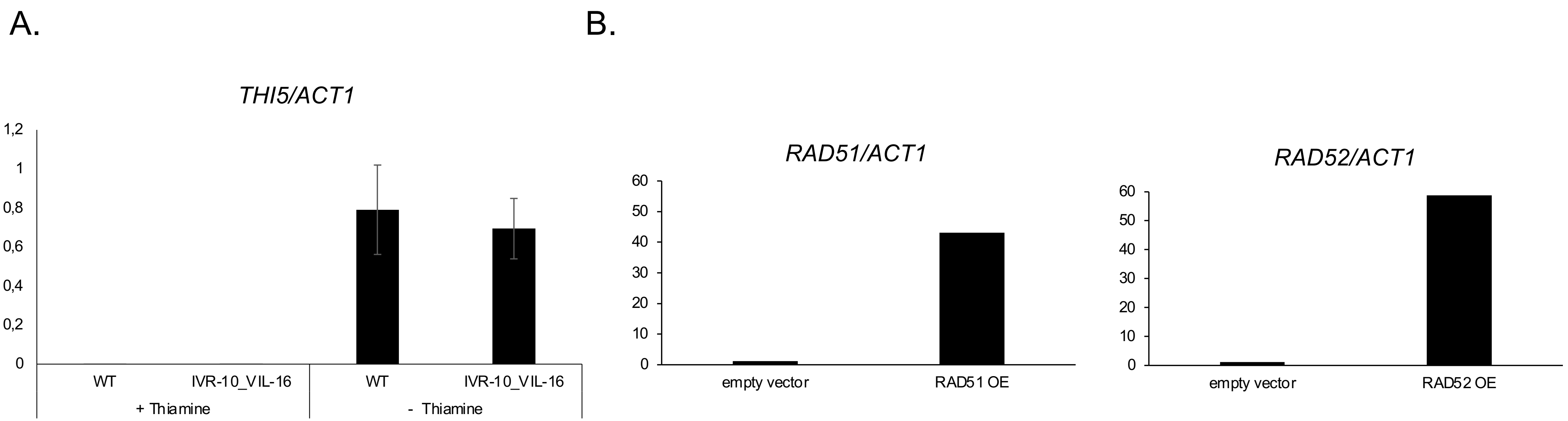

S4 Fig: Quantification of mRNA levels by RT-qPCR using *ACT1* for normalization. **A**. Relates to Fig 4A. IVR-10_VIL-16 is the translocated strain containing *THI5* translocated downstream of the IVR-10 locus. Mean ratios of *THI5* and *ACT1* RT-qPCR quantifications from two independent experiments are indicated. Error bars indicate the deviation from the mean. **B**. Relates to Fig 5B. Ratios of *RAD51* and *RAD52* RT-qPCR quantifications normalized by the RT-qPCR quantification of *ACT1*. Overexpression of *RAD51* and *RAD52* was verified with one experiment only.
