# Supplementary material for: Comprehensive analysis of cis- and trans-acting factors affecting ectopic Break-Induced Replication
Source: PLoS Genet. 2022 Jun 21;18(6):e1010124. doi: 10.1371/journal.pgen.1010124 (PMC9249352; doi:10.1371/journal.pgen.1010124)
Supplement: S1 Table — (DOCX) [file pgen.1010124.s005.docx]

**S1 Table. List of strains**

| strain number | genotype | origin |
| --- | --- | --- |
| BY4741 | *MAT****a*** *his3∆1 leu2∆0 met15∆0 ura3∆0* | [1] |
| BY4742 | *MAT𝛼 his3∆1 leu2∆0 lys2∆0 ura3∆0* | [1] |
|  | *MAT****a*** *his3∆1 leu2∆0 met15∆0 ura3∆0* ***ydr541c::KANMX4* (11kb from TEL04R)^#^** | [1] |
|  | *MAT****a*** *his3∆1 leu2∆0 met15∆0 ura3∆0* ***ydr479c::KANMX4* (135kb from TEL04R) ^#^** | [1] |
|  | *MAT****a*** *his3∆1 leu2∆0 met15∆0 ura3∆0* ***ydr443c::KANMX4* (182kb from TEL04R) ^#^** | [1] |
|  | *MAT****a*** *his3∆1 leu2∆0 met15∆0 ura3∆0* ***ydr387c::KANMX4* (282kb from TEL04R) ^#^** | [1] |
|  | *MAT****a*** *his3∆1 leu2∆0 met15∆0 ura3∆0* ***ydr338c::KANMX4* (383kb from TEL04R) ^#^** | [1] |
|  | *MAT****a*** *his3∆1 leu2∆0 met15∆0 ura3∆0* ***ydr294c::KANMX4* (480kb from TEL04R) ^#^** | [1] |
|  | *MAT****a*** *his3∆1 leu2∆0 met15∆0 ura3∆0* ***ydr198c::KANMX4* (378kb from TEL04R) ^#^** | [1] |
|  | *MAT****a*** *his3∆1 leu2∆0 met15∆0 ura3∆0* ***ydr146c::KANMX4* (781kb from TEL04R) ^#^** | [1] |
|  | *MAT****a*** *his3∆1 leu2∆0 met15∆0 ura3∆0* ***ydr101c::KANMX4* (875kb from TEL04R) ^#^** | [1] |
|  | *MAT****a*** *his3∆1 leu2∆0 met15∆0 ura3∆0* ***ydr046c::KANMX4* (81kb from TEL04R) ^#^** | [1] |
|  | *MAT****a*** *his3∆1 leu2∆0 met15∆0 ura3∆0* ***ydr001c::KANMX4* (1080kb from TEL04R) ^#^** | [1] |
| BLY344 | *MAT𝛼 his3∆1 leu2∆0 lys2∆0 ura3∆0* ***pol32::HPH*** | This study |
| BLY1183 | *MAT****a*** *his3∆1 leu2∆0 met15∆0 ura3∆0 ura3∆::NATMX* ***pif1m2*** | This study |
| BLY1487 | *MAT****a*** *his3∆1 leu2∆0 met15∆0 ura3∆0* ***pol32::HPH*** *ura3∆::NATMX* ***pif1m2*** | Gift from V. Borde |
| BLY1218 | *MAT****a*** *his3∆1 leu2∆0 met15∆0 ura3∆0* ***sir3::kanMX*** | This study |
| BLY1491 | *MAT****a*** *his3∆1 leu2∆0 met15∆0 ura3∆0* ***dun1::HPH*** | This study |
| BLY1493 | *MAT****a*** *his3∆1 leu2∆0 met15∆0 ura3∆0* ***sml1::HPH*** | This study |
| BLY1552 | *MAT****a*** *his3∆1 leu2∆0 met15∆0 ura3∆0* ***dun1::HPH sml1::kanMX*** | This study |
| BLY433 | *MAT****a*** *his3∆1 leu2∆0 met15∆0 ura3∆0* ***mph1::kanMX*** | This study |
| BLY1575 | *MAT****a*** *his3∆1 leu2∆0 met15∆0 ura3∆0* ***srs2::kanMX*** | [1] |
| BLY1575_1 | *MAT𝛼 his3∆1 leu2∆0 lys2∆0 ura3∆0* ***srs2::kanMX*** | This study |
| BLY1598 | *MAT****a/****MAT𝛼 his3∆1/his3∆1 leu2∆0/leu2∆0 met15∆0/MET15 LYS2/lys2∆0 ura3∆0/ura3∆0* ***srs2::kanMX/srs2::kanMX*** | BLY1575 x BLY1575_1 |
| BLY1597 | ***MATa::NAT/****MAT𝛼  his3∆1/his3∆1 leu2∆0/leu2∆0 met15∆0/MET15 LYS2/lys2∆0 ura3∆0/ura3∆0* ***srs2::kanMX/srs2::kanMX*** | This study |
| BLY1576 | *MAT****a*** *his3∆1 leu2∆0 met15∆0 ura3∆0*  Translocation IVR-10_VIIR-101 | This study |
| BLY1577 | *MAT****a*** *his3∆1 leu2∆0 met15∆0 ura3∆0*  Translocation IVR-113_XVIR-12 (No LTR)* | This study |
| BLY1620 | *MAT****a*** *his3∆1 leu2∆0 met15∆0 ura3∆0*  Translocation IVR-10_VIIR-291 | This study |
| BLY1622 | *MAT****a*** *his3∆1 leu2∆0 met15∆0 ura3∆0* ***srs2::kanMX***  Translocation IVR-113_XVIR-10 (No LTR)* | This study |
| BLY1651 | *MAT****a*** *his3∆1 leu2∆0 met15∆0 ura3∆0*  Translocation IVR-10_VIR-10 (No Y’ element)* | This study |
| BLY1656 | *MAT****a*** *his3∆1 leu2∆0 met15∆0 ura3∆0* ***srs2::kanMX***  Translocation IVR-10_VIR-10 (No Y’ element)* | This study |
| BLY1639 | *MAT****a*** *his3∆1 leu2∆0 met15∆0 ura3∆0*  Translocation IVR-113_VIR-10 (No LTR, No Y’ element)* | This study |
| BLY1654 | *MAT****a*** *his3∆1 leu2∆0 met15∆0 ura3∆0* ***srs2::kanMX***  Translocation IVR-113_VIR-10 (No LTR, No Y’ element)* | This study |
| BLY1599 | *MAT****a*** *his3∆1 leu2∆0 met15∆0 ura3∆0*  Translocation IVR-10_VIL-16** |  |

**^#^** Strain used to initiate BIR from the *KANMX4* cassette, whose distance from the right telomere of chromosome IV TEL04R) is given in parentheses

* “No LTR” and “no Y’ element” refers to the absence of LTR or Y’ element in the chromosome fragment used as BIR template

** VIL chromosome fragment contains the thiamine regulon composed of *SNO3*, *SNZ3* and *THI5*

**Reference**

1. Winzeler EA, Shoemaker DD, Astromoff A, Liang H, Anderson K, Andre B, et al. Functional characterization of the S. cerevisiae genome by gene deletion and parallel analysis. Science. 1999;285: 901–906. doi:10.1126/science.285.5429.901
