# Supplementary material for: Comprehensive analysis of cis- and trans-acting factors affecting ectopic Break-Induced Replication
Source: PLoS Genet. 2022 Jun 21;18(6):e1010124. doi: 10.1371/journal.pgen.1010124 (PMC9249352; doi:10.1371/journal.pgen.1010124)
Supplement: S2 Table — (DOCX) [file pgen.1010124.s006.docx]

**S2 Table. Oligonucleotides used to generate reciprocal translocations**

| **BLY1576: Reciprocal translocation IVR-10_VIIR-101** | | | |
| --- | --- | --- | --- |
| **Name** | **Sequence 5’ – 3’** | **Type** | **Description** |
| TU002 | TCGAACGCTCTTCAATCAACTACTATCTTACTTCCACGTTTTAGAGCTAGAAATAGCAAG | gRNA | gRNA targeting the IVR-10 locus |
| TU003 | CTAGACGCTCTTCAAACTTTTTGTTGGAATGTTAAGCGATCATTTATCTTTCACTGCGG | gRNA | gRNA targeting the VIIR01 locus |
| TU010a | AAATATTTAATTGCATGTTATTTTGTTTAACTACTATCTTACTTCAAAAGGAAAAATAAAACAATAATTTCGTTCCCCATCTATCCTTCG | donor | 45bp of IVR-10 locus + 45bp of VIIR01 locus |
| TU010b | CGAAGGATAGATGGGGAACGAAATTATTGTTTTATTTTTCCTTTTGAAGTAAGATAGTAGTTAAACAAAATAACATGCAATTAAATATTT | donor | reverse complement of TU010a |
| TU011a | AGTAGAGCAGATCTTTTAGCAAGGATCCGCTTAACATTCCAACAACACTGGTCCACCGGACCCTCAGGCTTCCTTGCCTTGTGGAACCAG | donor | 45bp of IVR-10 locus + 45bp of IVR-10 locus |
| TU011b | CTGGTTCCACAAGGCAAGGAAGCCTGAGGGTCCGGTGGACCAGTGTTGTTGGAATGTTAAGCGGATCCTTGCTAAAAGATCTGCTCTACT | donor | reverse complement of TU011a |
| TU014 | AGGCAATGTTCGTGAGTAAGT | translocation validation | forward IVR-10 locus |
| TU015 | TCATGTGCCAAGGTTTTGTGT | translocation validation | reverse IVR-10 locus |
| TU016 | TACCGCTGTTGATACCAAGGA | translocation validation | forward VIIR01 locus |
| TU017 | TAAAAAGCACGGAAGGAACGC | translocation validation | reverse VIIR01 locus |
|  | |  |  |
| **BLY1577: Reciprocal translocation IVR-113_XVIR-12 (No LTR, presence of Y' element)** | | | |
| **Name** | **Sequence 5’ – 3’** | **Type** | **Description** |
| TU004 | TCGAACGCTCTTCAATCGCGATACAGTACGTGTCATGGTTTTAGAGCTAGAAATAGCAAG | gRNA | gRNA targeting the IVR-113 locus |
| TU005 | CTAGACGCTCTTCAAACTCAGTGTCAGCATGAGTATTGATCATTTATCTTTCACTGCGG | gRNA | gRNA targeting the XVIR-12 locus |
| TU012a | AAAACTCGCCTGCACTCCGAAACGAAATGCGATACAGTACGTGTCTGAAGGATTATTGGTATTGGCCGGTTGTGTTTTTTCTATACATAG | donor | 45bp of IVR-113 locus + 45bp of XVIR-12 locus |
| TU012b | CTATGTATAGAAAAAACACAACCGGCCAATACCAATAATCCTTCAGACACGTACTGTATCGCATTTCGTTTCGGAGTGCAGGCGAGTTTT | donor | reverse complement of TU012a |
| TU013a | GGGCTTACAGCTCCTATGTGGATATGTGAATACTCATGCTGACACATGCGGTTAGTATATATACATATATATATATATATATATATTCAA | donor | 45bp of XVIR-12 locus + 45bp of IVR-113 locus |
| TU013b | TTGAATATATATATATATATATATATGTATATATACTAACCGCATGTGTCAGCATGAGTATTCACATATCCACATAGGAGCTGTAAGCCC | donor | reverse complement of TU013a |
| TU018 | ACCACAACGGAGACCCAATC | translocation validation | forward IVR-113 locus |
| TU019 | CGAATACTACCATGAGTTGACCA | translocation validation | reverse IVR-113 locus |
| TU020 | TACTGTTTTGTGGGTTGGGAT | translocation validation | forward XVIR-12 locus |
| TU021 | ATGGGTCCAGCGTACAACTC | translocation validation | reverse XVIR-12 locus |
| **BLY1620: Reciprocal translocation IVR-10_VIIR-291** | | | |
| **Name** | **Sequence 5’ – 3’** | **Type** | **Description** |
| TU002 | TCGAACGCTCTTCAATCAACTACTATCTTACTTCCACGTTTTAGAGCTAGAAATAGCAAG | gRNA | gRNA targeting the IVR-10 locus |
| TU105 | CTAGACGCTCTTCAAACACCTTTCCAACATGTCGCTGGATCATTTATCTTTCACTGCGG | gRNA | gRNA targeting the VIIR-291 locus |
| TU107 | AAATATTTAATTGCATGTTATTTTGTTTAACTACTATCTTACTTCGGTCGGGCAGTAAAGCAGAATTTCAGAAATACTAGTACGATTGTT | donor | 45bp of IVR-10 locus + 45bp of VIIR-291 locus |
| TU108 | AACAATCGTACTAGTATTTCTGAAATTCTGCTTTACTGCCCGACCGAAGTAAGATAGTAGTTAAACAAAATAACATGCAATTAAATATTT | donor | reverse complement of TU107 |
| TU109 | TACCCGACTCTGAAGAAAAACAAAAAAACAGCGACATGTTGGAAACACTGGTCCACCGGACCCTCAGGCTTCCTTGCCTTGTGGAACCAG | donor | 45bp of VIIR-291 locus + 45bp of IVR-10 locus |
| TU110 | CTGGTTCCACAAGGCAAGGAAGCCTGAGGGTCCGGTGGACCAGTGTTTCCAACATGTCGCTGTTTTTTTGTTTTTCTTCAGAGTCGGGTA | donor | reverse complement of TU109 |
| TU014 | AGGCAATGTTCGTGAGTAAGT | translocation validation | forward IVR-10 locus |
| TU015 | TCATGTGCCAAGGTTTTGTGT | translocation validation | reverse IVR-10 locus |
| TU115 | TAGCGCCTATACGAAGCAGC | translocation validation | forward VIIR-291 locus |
| TU116 | TCTAATCAATGCAACGTGTTTGAC | translocation validation | reverse VIIR-291 locus |
|  | | | |
| **BLY1639: Reciprocal translocation IVR-113_VIR-10 (No LTR, No Y' elements)** | | | |
| **Name** | **Sequence 5’ – 3’** | **Type** | **Description** |
| TU004 | TCGAACGCTCTTCAATCGCGATACAGTACGTGTCATGGTTTTAGAGCTAGAAATAGCAAG | gRNA | gRNA targeting the IVR-113 locus |
| TU127 | CTAGACGCTCTTCAAACATTGTCAATGAGCAGGTTGAGATCATTTATCTTTCACTGCGG | gRNA | gRNA targeting the VIR-10 locus |
| TU140 | AAAACTCGCCTGCACTCCGAAACGAAATGCGATACAGTACGTGTCAATTGGATTATTTTTGGCTGTTCAAGATTAAAACAGACTCAGTAG | donor | 45bp of the IVR-113 locus + 45bp of the VIR-10 locus |
| TU141 | CTACTGAGTCTGTTTTAATCTTGAACAGCCAAAAATAATCCAATTGACACGTACTGTATCGCATTTCGTTTCGGAGTGCAGGCGAGTTTT | donor | reverse complement of TU140 |
| TU142 | ATGTCCATCAAGACTAGCGTAATAAAAATCAACCTGCTCATTGACATGCGGTTAGTATATATACATATATATATATATATATATATTCAA | donor | 45bp of the VIR-10 locus + 45bp of the IVR-113 locus |
| TU143 | TTGAATATATATATATATATATATATGTATATATACTAACCGCATGTCAATGAGCAGGTTGATTTTTATTACGCTAGTCTTGATGGACAT | donor | reverse complement of TU142 |
| TU018 | ACCACAACGGAGACCCAATC | translocation validation | forward IVR-113 locus |
| TU019 | CGAATACTACCATGAGTTGACCA | translocation validation | reverse IVR-113 locus |
| TU132 | ACACACAGAGACCACCGTTTA | translocation validation | forward VIR-10 locus |
| TU133 | AGCCAGGGTATCTTCTGTGAG | translocation validation | reverse VIR-10 locus |
|  | | | |
| **BLY1599: Reciprocal translocation IVR-10_VIL-16. Locates *SNO3*, *SNZ3* and *THI5* thiamine regulon on chromosome IV.** | | | |
| **Name** | **Sequence 5’ – 3’** | **Type** | **Description** |
| TU002 | TCGAACGCTCTTCAATCAACTACTATCTTACTTCCACGTTTTAGAGCTAGAAATAGCAAG | gRNA | gRNA targeting the IVR-10 locus |
| TU081 | CTAGACGCTCTTCAAACGTTCTCAAATACAGTGCTAAGATCATTTATCTTTCACTGCGG | gRNA | gRNA targeting the VIL-16 locus |
| TU087 | AAATATTTAATTGCATGTTATTTTGTTTAACTACTATCTTACTTCCTCAAATACAGTGCTAATAATCACATGGTTCGTAGAGGGCGTGAA | donor | 45bp of the IVR-10 locus + 45bp of the VIL-16 locus |
| TU088 | TTCACGCCCTCTACGAACCATGTGATTATTAGCACTGTATTTGAGGAAGTAAGATAGTAGTTAAACAAAATAACATGCAATTAAATATTT | donor | reverse complement of TU087 |
| TU089 | CTGGTTCCACAAGGCAAGGAAGCCTGAGGGTCCGGTGGACCAGTGAACAGGTCGGTGTATCATTTTCTACAATTTTGTACATAGAAAATT | donor | 45bp of the VIL-16 locus + 45bp of the IVR-10 locus |
| TU090 | AATTTTCTATGTACAAAATTGTAGAAAATGATACACCGACCTGTTCACTGGTCCACCGGACCCTCAGGCTTCCTTGCCTTGTGGAACCAG | donor | reverse complement of TU089 |
| TU014 | AGGCAATGTTCGTGAGTAAGT | translocation validation | forward IVR-10 locus |
| TU015 | TCATGTGCCAAGGTTTTGTGT | translocation validation | reverse IVR-10 locus |
| TU091 | GCAAGGCAAGCAGATGCAAT | PCR trans. validation | forward VIL-16 locus |
| TU092 | ACGTGGTATAATAGGCTCAAGGA | PCR trans. validation | reverse VIL-16 locus |
