# Supplementary material for: Comprehensive analysis of cis- and trans-acting factors affecting ectopic Break-Induced Replication
Source: PLoS Genet. 2022 Jun 21;18(6):e1010124. doi: 10.1371/journal.pgen.1010124 (PMC9249352; doi:10.1371/journal.pgen.1010124)
Supplement: S3 Table — All CFVs except pLS192 and pBL003 are designed to initiate BIR from chromosome IV regions, whose coordinates are from the Saccharomyces Genome Database (SGD). pLS192 is designed to initiate BIR from the left arm of chromosome III [51]. pBL003 contains the KANMX4 cassette amplified from the BY4741 rad27::KANMX4 strain using the U2 and D2 oligonucleotides [31] (http://www-sequence.stanford.edu/group/yeast_deletion_project/PCR_strategy.html). The resulting CFV is designed to initiate BIR from any KANMX4 cassette whose transcription orientation is from the telomere to the centromere. BamHI (GGATCC) and BglII (AGATCT) sites are underlined. Note that the regions amplified to construct pBL013 and pBL014 contain a BamHI and a BglII site, respectively. (DOCX) [file pgen.1010124.s007.docx]

**S3 Table. Chromosome fragmentation vectors (CFVs) used**

| Plasmid name | Distance from telomere (kb) | Chromosome coordinates of BIR initiating region | Oligonucleotides sequences used to amplify the corresponding genomic loci and clone them into  EcoNI-BglII double digested pLS192 |
| --- | --- | --- | --- |
| CA50 | 166 | 1360939 - 1365875 | TACAGGGTTTCAGGGATAAATGGA  TGTCTAGATCTCTGCTAAGGTGTTTGTTACCCGA |
| pBL013 | 117 | 1409510 - 1414996 | TTGTCTCCAATTGGCATACG  TTCCCGCTGCTGGTACTATT |
| CAG | 99 | 1428154 - 1433026 | CCAATCTTGGCTTCCTCCTA  TCAAGGATCCAATGCAGAGGCGATGAAGC |
| CAM | 57 | 1469940 - 1474882 | ATCTCCGTTAAATGAGCAAG  TCATGGATCCGTACGAATTGAAATGAAATGT |
| TU480 | 46 | 1480926 - 1485534 | GATGCCAGCCATCATGTCAA  TCGAACGGATCCATGCATGAGTTTTTGTTTTCCATTC |
| TU485 | 41 | 1485838 - 1490734 | TCTCTGCTTGAGCATTTGGG  TCGAACGGATCCTCAGACCAGGGAAAAACCCT |
| TU491 | 36 | 1491010 - 1495847 | ATCGCTGTCGGCTAAGTAAA  TCGAACGGATCCGTGCAGAATAACTTGGCGAAAA |
| CAD | 26 | 1500792 - 1505691 | CTGGAAGATATTGTTTATCCG  TGTCTAGATCTCATTATTTTAATCCTTATATTTCCA |
| pBL014 | 10 | 1517068 - 1521977 | AAGGTTTTCCTGTGGTAGTTTC  AGTCAGTGTGACCCAATAAGAA |
| pLS192 | 97 | 96821-102096 |  |
| pBL003 |  | *KANMX4* | U2 : cgtacgctgcaggtcgac  D2 : atcgatgaattcgagctcg |

All CFVs except pLS192 and pBL003 are designed to initiate BIR from chromosome IV regions, whose coordinates are from the *Saccharomyces* Genome Database (SGD). pLS192 is designed to initiate BIR from the left arm of chromosome III [1]. pBL003 contains the *KANMX4* cassette amplified from the BY4741 *rad27*::*KANMX4* strain using the U2 and D2 oligonucleotides [2] (<http://www-sequence.stanford.edu/group/yeast_deletion_project/PCR_strategy.html>). The resulting CFV is designed to initiate BIR from any *KANMX4* cassette whose transcription orientation is from the telomere to the centromere.

BamHI (GGATCC) and BglII (AGATCT) sites are underlined. Note that the regions amplified to construct pBL013 and pBL014 contain a BamHI and a BglII site, respectively.

**References**

1. Marrero VA, Symington LS. Extensive DNA End Processing by Exo1 and Sgs1 Inhibits Break-Induced Replication. Maizels N, editor. PLoS Genet. 2010;6: e1001007. doi:10.1371/journal.pgen.1001007

2. Winzeler EA, Shoemaker DD, Astromoff A, Liang H, Anderson K, Andre B, et al. Functional characterization of the S. cerevisiae genome by gene deletion and parallel analysis. Science. 1999;285: 901–906. doi:10.1126/science.285.5429.901
