# Supplementary material for: Comprehensive analysis of cis- and trans-acting factors affecting ectopic Break-Induced Replication
Source: PLoS Genet. 2022 Jun 21;18(6):e1010124. doi: 10.1371/journal.pgen.1010124 (PMC9249352; doi:10.1371/journal.pgen.1010124)
Supplement: Attachment — Submitted filename: rebuttal_V3.docx [file pgen.1010124.s009.docx]

Reviewer #1: This new work demonstrates that break induced replication (BIR) is more efficient when strand invasion occurs closer to the telomere. This is shown using many new recombination assays on different chromosomes. A number of additional features and proteins dictates the efficiency of BIR like transcription, chromatin compaction, Mph1, the level of Rad51 and Rad52 and other. Altogether this is very elegant and well written study. After addressing questions listed below it should be considered for publication.

1. The major finding of this work is that BIR is more efficient when strand invasion occurs close to the telomere. What is not clear is whether the proximity to telomere and telomere specific features or only shorter DNA synthesis improve the efficiency of BIR?

A key result is the good fit of BIR efficiency as a function of the length of DNA to synthesize with two exponential functions. Such a fit supports a mechanistic model with two DNA synthesis modes taking place successively during BIR, from which we can extract the corresponding apparent processivity parameters. This model does not involve any telomere specific feature. Therefore, in the absence of indications supporting a specific influence of telomeric sequences on BIR efficiency we parsimoniously consider that in our case BIR efficiency is only determined by the length of DNA to synthesize.

Although this model still has to be experimentally demonstrated, the two successive DNA synthesis modes are in line with what we previously proposed in collaboration with Lorraine Symington (Smith et al 2007), and is further supported by recent work from Anna Malkova’s lab showing that leading strand synthesis during BIR cannot proceed past 30 kb without primase activity (Liu et al 2021).

2. The limitation of this work is that they look at ectopic BIR only (limited homology). With longer homology as in “allelic” BIR between truncated chromosome III and full size chromosome III as used by Malkova or Haber labs, BIR is nearly 100% efficient with the distance of over 100 kb to the telomere. It would be interesting for the authors to design one allelic assay and test whether BIR far away from chromosome ends increases when there is no homology limitation. Also the title and abstract should state that what is studied here is ectopic BIR and not BIR in general unless they study allelic BIR in a revised manuscript.

We acknowledge that this is a major difference between the two experimental systems for which we do not have a definitive explanation. Our experimental system relies on the transformation of a BIR substrate to initiate ectopic BIR. Hence, several parameters could be responsible for this difference in BIR efficiency. Interestingly, the Haber lab (Lydeard et al., 2007) and the Symington lab (Donnianni and Symington, 2013) also previously used ectopic BIR assays relying on the induction of a HO DSB within a native yeast chromosome. In both cases BIR efficiency was below 100% and a negative effect of the length of DNA that BIR must copy was observed on BIR efficiency. In conclusion, it seems that only allelic BIR shows 100% efficiency. This property requires further investigation to understand the underlying mechanism that may promote more favorable conditions for BIR between homologous chromosomes than between non-homologous chromosomes. A short paragraph addressing this point was added in the discussion.

Establishing and testing a novel allelic assay was not compatible with the timeframe of this revision, so we revised the title, the abstract and the text accordingly. Note that the distinction between allelic and ectopic BIR has never been done so far while many reports focused on ectopic BIR.

3. Rad51 and Rad52 overexpression needs to be documented by Western blots. It is important to know the fold increase in protein level. Alternatively, in case previous studies showed protein fold increase using the same multicopy plasmids, it would need to be stated.

The pRS423:RAD51 overexpression plasmid is from the Symington lab (Davis and Symington 2001). The pRS423:RAD52 overexpression plasmid is from the Patrick Sung lab and was shown to lead to a 20-fold over-expression (Sung 1997). These are two-micron plasmids that were subsequently used by the Symington lab to show BIR defect suppression of *rfa1* mutants (Ruff et al. 2016). Although we agree that Western blots are the best way to show protein overexpression, they are rarely shown to support overexpression from such two-micron plasmids (*i.e.* Davis and Symington, 2001, Lydeard et al., 2010, Ruff et al., 2016). As a proxy, we provided qPCR quantification of the corresponding mRNAs (Figure S4) that clearly supports similar overexpression of *RAD51* and *RAD52*. The fact that Rad52 was 20-fold overexpressed under such conditions (Sung 1997), suggests that Rad51 is overexpressed to a similar extent.

4. Page 10 lane 1-2

“BIR events depend on POL32 and to a lesser extend on PIF1”

For this conclusion one would need to perform analysis in pif1delta not the hypomorphic mutant pif1-m2 that still partially localizes to nucleus. Therefore relative contribution of Pol32 and Pif1 to BIR cannot be estimated with present data. One set of experiments should be done in pif1delta or conclusion should be modified.

Because pif1delta is also associated with a “petite phenotype”, we preferred to simply modify the conclusion accordingly.

Minor
In discussion of the biphasic nature of BIR it is important to remember that BIR remains extremely mutagenic even 90 kb from strand invasion site, and strands are inherited in conservative manner likely all the way to the end of chromosome. This suggests that at least the basic mechanism of BIR is the same within the first 10-20 kb and 100 kb from strand invasion.

Starting from Smith et al., 2007, followed by Liu et al., 2021 and including our present data, there is strong support for a biphasic nature of DNA synthesis during BIR. The fact that Malkova and collaborators (Elango et al., 2019) have observed similar, high BIR-associated mutation rates for a *ura3-29* base substitution reporter inserted at positions located 0 kb, 16 kb, and 90 kb away from the BIR initiation site suggests that the two replication modes are similarly mutagenic. We therefore included the following sentence in the discussion: “The fact that Malkova and collaborators have observed similar, high BIR-associated mutation rates for a *ura3-29* base substitution reporter inserted at positions located 0 kb, 16 kb, and 90 kb away from the BIR initiation site suggests that both replication phases of BIR would be similarly mutagenic (Elango et al., 2019).”

Reviewer #2: Llorente paper

This is a fine paper exploring the effect of template length and other factors on successful break-induced replication. The authors find that BIR copying the terminal 10-20 kb is much more successful than events that must copy a longer distance. Some of this has been inferred from a few data points previously, but the present MS is a careful analysis of the problem.

1. There is one intrinsic contradiction to this analysis that the authors should address. In a disome or diploid in which a truncated chromosome III is cleaved and repaired by BIR, the efficiencies of copying 100 kb are quite high (Malkova et al., 2005; Liu et al. 2021). Is this a feature of the fact that the broken chromosome is a pre-existing chromosome and not a fragment just introduced by transformation?

Similar question and answer as question 2 from reviewer 1.

2. Two different normalizations are used in the paper: the repair of a BUD3-containing reference chromosome fragment or the co-transformation of a circular plasmid.

2.1. If the BUD3 is marked by the same URA3 as the test fragment, does this mean they come intrinsically from different experiments? Is this a problem, given the high variability of the outcomes in Fig. 2 (from 18% to 52%)? I can’t figure out how many different locations contribute to this big spread. Maybe they could be discriminated in some way (circles, squares, x, etc)?
Is BUD3 (100 kb from a telomere) an intrinsically low reference point? What’s the ratio between BUD3 repair and transformation with the circle?

2.2. What’s the marker on the reference circular plasmid?

The use of *BUD3* as a BIR initiating point is restricted to the experiments shown in Figure 1 . The rationale for using BIR initiated at *BUD3* as a normalization strategy in Figure 1 is linked to the facts that the strains used to initiate BIR from the *KANMX4* cassette are all knockout mutants (where *KANMX4* is the knockout marker) and that these knockout mutations could have unexpected effects on BIR efficiency. Normalizing using BIR initiated at *BUD3* allowed to correct for any possible effect on BIR of the mutations, which is not the case when a circular plasmid is used to normalize.

The experimental system is the same as in Davis and Symington 2004, and *BUD3* is one of the BIR initiating loci they used without any particular property. Using this system, BIR efficiency is classically expressed as the number of transformants with a linear plasmid divided by the number of transformants with a corresponding circular plasmid, both containing *URA3* as a selection marker. The mean efficiency for BIR initiated at *BUD3* was 6.3 +/- 4%. This is similar to the BIR efficiency shown in Figure 2 for the BIR initiating locus located 99 kb away from TEL04R. Importantly, each x-axis point from Figure 2B corresponds to a unique BIR initiating locus described in Figure 2A, which is why all BIR efficiency points are represented by a unique filled circle symbol. The rather high variability in BIR efficiency present in our experiments is intrinsic to the assay system and was also observed in Davis and Symington 2004, the first paper to use it. The fit with the exponential functions that we provide takes into account this variability.

3 p.6. data not shown… show it in a supplement. Are these data in the curve fitting?

These data were not used for the curve fitting: the experimental points shown in Figure 2B correspond exclusively to data from chromosome IV. These data being accessory, we simply do not refer to them anymore in the manuscript.

4. The authors refer to repeated cycles of initiation, but isn’t this instability confined to the initial 20 or 30 kb? Later the authors suggest (as first suggested by Lydeard et al and then by Liu et al) that Polε may take over later in the synthesis. This seems reasonable.

At each step of DNA synthesis there is a non-zero probability, however low, that the polymerase dissociates prematurely, which leads either to a re-initiation phase or to synthesis termination. This kind of process is expected to result in a negative correlation between BIR efficiency and the length of DNA to synthesize that takes the form of decreasing exponential functions. In case the probability of polymerase dissociation would be extremely low, the fitting exponential function would look like a constant function. However, we observed that BIR efficiency as a function of DNA length to synthesize is well fitted by a combination of two clearly decreasing exponential functions, which strongly suggests a significant polymerase instability even after the synthesis of the first 35-40 kb of DNA (second exponential fitting curve).

5. What to AIC numbers mean? How do they relate to confidence intervals?

Akaike Information Criterion (AIC) provides a measure of the fit of a model to a set of data. Given a set of candidate models for the data, AIC evaluates them by taking into account both their likelihood functions and their numbers of estimated parameters. Indeed, AIC rewards goodness of fit (as assessed by the likelihood function) and penalizes the number of parameters, thus discouraging overfitting. For a given set of models, the preferred model is the one with the lowest AIC value. The first formal presentation of AIC is in Akaike, H. (1974) ‘A new look at the statistical model identification’, IEEE Transactions on Automatic Control, 19(6), pp. 716–723. doi:10.1109/TAC.1974.1100705.

AIC does not relate to confidence intervals.

6. p. 9 I am confused by this sentence: the probability of BIR reactions to be irreversibly
disrupted at each kb decreases after the synthesis of ~35-40 kb of DNA by a factor of
13, from 0.065 (1-0.935) to 0.005 (1-0.995). This required me to read it several times. Better:
“Over the first 35-40 kb, probability that BIR will be irreversibly disrupted is 0.065/kb , after which the probability falls by a factor of 13, to 0.005/kb.”
But, still, this seems hard to understand. If p(disruption) is 0.065/kb, then after 30 kb, is p = 1.95? So what am I missing? The decrease must not be a linear function. A careful description is needed.

The wording of this part has been clarified according to the reviewer’s indications.

As the reviewer says, the probability that DNA synthesis is irreversibly disrupted before 30 kb (for example) cannot be simply computed by multiplying *p_1_* = 0.065/kb by 30, the number of kb. This computation is in fact quite complex, as detailed below.

The probability that DNA synthesis is irreversibly disrupted before 30 kb is equal to the probability that DNA synthesis produces 0 kb DNA and stops immediately, plus the probability that DNA synthesis produces 1 kb DNA and then stops, plus the probability that DNA synthesis produces 2 kb DNA and then stops, plus the probability that DNA synthesis produces 3 kb DNA and then stops, etc., plus the probability that DNA synthesis produces 29 kb DNA and then stops.

The probability that DNA synthesis produces *x* kb DNA and then stops is equal to *k* (the probability that BIR is initiated at the DSB end) multiplied by *p_1_* (the probability that DNA synthesis goes on for 1 kb) to the power of *x* since *x* kb are produced, multiplied by (1-*p_1_*), the probability that synthesis is then irreversibly disrupted, which gives :

*k*(1-*p_1_*)*p_1_*^x^.

The probability that DNA synthesis is irreversibly disrupted before 30 kb is thus equal to the sum :

*k*(1-*p_1_*)*p_1_^0^*.+ *k*(1-*p_1_*)*p_1_^1^*.+ *k*(1-*p_1_*)*p_1_^2^*.+ *k*(1-*p_1_*)*p_1_^3^*.+ … + *k*(1-*p_1_*)*p_1_^29^*

*= k*(1*-p_1_*)(1*+p_1_+p_1_^2^+p_1_^3^+…+p_1_^29^*)

*= k*(1*-p_1_*)(1*-p_1_^30^*)/(1*-p_1_*)

(since as a general rule we have : 1+*x*+*x^2^*+…+*x^n^* = (1-*x^n+1^*)/(1-*x*))

*= k*(1*-p_1_^30^*).

The computation of the probability that DNA synthesis starts but is irreversibly disrupted before 30 kb is thus complicated, which is one of the reasons why in the article we have presented the computation of the probability of the complementary event, that is the probability that DNA synthesis starts and proceeds as far as *x* kb, which corresponds to BIR efficiency.

7. Whether the effect of deleting Sir3 is on “accessibility” isn’t so clear to me. In MAT switching, where the donor is heterochromatic, there is very little effect on deleting sir3 on donor usage. Most relevant, Tasponina and Haber (2014) found that deleting Sir3 had no significant effect on template switching, which required a “jump” into the donor sequence (heterochromatic or not).
It might be useful to consult the Saccharomyces genome database to see the effect of sir mutations on the level of transcription of sub-telomeric genes.

About the *sir3* phenotype, we wrote: “This is compatible with heterochromatin impairing BIR downstream and close to the BIR initiation locus only, but not when BIR events are initiated farther away.” So we are not referring to accessibility, but to chromatin structure that would be less permissive to BIR-associated DNA synthesis.

8. p. 12 Dun1 does considerably more than regulate dNTP levels. Could its effect be related to changing the DNA damage response and to the levels of some HR or chromatin proteins?

It is definitely a possibility. We modified the text accordingly: “On the contrary, in the absence of Dun1, *which has many functions in response to DNA damage* including Sml1 degradation and therefore up-regulation of the ribonucleotide reductase activity,”

9. p. 15 Interchromosomal jumps between solo delta elements was previously shown by Anand et al. (2014).

Anand et al. (2014) has been added to Smith et al 2007 as references to interchromosomal jumps between solo delta elements.

10. p. 17 define GCR

done

11. I am not clear how the authors’ view of Srs2 differs from that of Malkova’s lab? The effect of ploidy is curious. I wonder if BIR would be different in G2/M cells where there was a sister chromatid. (I am speculating here but I want to be clear that I have no expectation that the authors should do this analysis for the present paper!)
It may be relevant that Ira et al. (2002) found that srs2Δ decreased HO-induced ectopic recombination without crossovers but somehow had less effect on outcomes involving crossovers. Or not… very confusing protein.

Srs2 role seems to be related to the presence of several copies of the same sequence in the nucleus. As stated in the text, the fact that we do not see any defect in haploid cells from asynchronous cultures could be explained by the fact that BIR could happen outside G2/M and that the presence of only two copies of the same sequence (one from the BIR template and one from the DNA molecule undergoing BIR) is not toxic in the absence of Srs2. The toxicity would appear when more than two copies of the same sequence are present, which is the case in G2/M haploid cells, in diploid cells, and close to telomeres.

Reviewer #3: The manuscript by Uribe-Calvillo et al describes a meticulous analysis of several of the properties that distinguish and define the BIR pathway. The authors made extensive use of cleverly designed BIR assays that included multiple engineered translocations to thoroughly characterize the already known negative relationship between BIR efficiency and length of the replication tract. The most valuable finding was the biphasic behaviour of the BIR efficiency plot where the authors were able to estimate a point where BIR likely transitions from short to long template extension modes, which possibly reflects a change in the replication machinery involved in the process. Overall, I thought this was a nice manuscript that will help move the BIR field forward in an appreciable way. The advancements reported in this work may not be huge or entirely novel, but the authors do provide important additional refinements on many of the key properties of BIR that had already been identified, but not explored in as much detail or in as much clarity as is provided here. Together, the various experiments reported should help better contextualize the instances where BIR is likely to happen in cells, relative to cases where chromosomal rearrangements (particularly non-reciprocal translocaitons) might look like BIR products, but are less likely to have actually arisen through BIR and thus were probably generated through cannonical 2-ended HR DSB repair.
I do not have any major concerns or suggestions that the authors would need to address.
